# Sneaking into the viral safe-houses: Implications of host components in regulating integrity and dynamics of rotaviral replication factories

**DOI:** 10.3389/fcimb.2022.977799

**Published:** 2022-09-14

**Authors:** Pritam Chandra, Shreya Banerjee, Priyanka Saha, Mamta Chawla-Sarkar, Upayan Patra

**Affiliations:** ^1^ Division of Virology, Indian Council of Medical Research National Institute of Cholera and Enteric Diseases, Kolkata, India; ^2^ Institute of Biochemistry II, Faculty of Medicine, Goethe University, Frankfurt, Germany

**Keywords:** rotavirus, replication, viroplasms, pro-viral and antiviral host determinants, host-directed antivirals

## Abstract

The biology of the viral life cycle essentially includes two structural and functional entities—the viral genome and protein machinery constituting the viral arsenal and an array of host cellular components which the virus closely associates with—to ensure successful perpetuation. The obligatory requirements of the virus to selectively evade specific host cellular factors while exploiting certain others have been immensely important to provide the platform for designing host-directed antiviral therapeutics. Although the spectrum of host-virus interaction is multifaceted, host factors that particularly influence viral replication have immense therapeutic importance. During lytic proliferation, viruses usually form replication factories which are specialized subcellular structures made up of viral proteins and replicating nucleic acids. These viral niches remain distinct from the rest of the cellular milieu, but they effectively allow spatial proximity to selective host determinants. Here, we will focus on the interaction between the replication compartments of a double stranded RNA virus rotavirus (RV) and the host cellular determinants of infection. RV, a diarrheagenic virus infecting young animals and children, forms replication bodies termed viroplasms within the host cell cytoplasm. Importantly, viroplasms also serve as the site for transcription and early morphogenesis of RVs and are very dynamic in nature. Despite advances in the understanding of RV components that constitute the viroplasmic architecture, knowledge of the contribution of host determinants to viroplasm dynamicity has remained limited. Emerging evidence suggests that selective host determinants are sequestered inside or translocated adjacent to the RV viroplasms. Functional implications of such host cellular reprogramming are also ramifying—disarming the antiviral host determinants and usurping the pro-viral components to facilitate specific stages of the viral life cycle. Here, we will provide a critical update on the wide variety of host cellular pathways that have been reported to regulate the spatial and temporal dynamicity of RV viroplasms. We will also discuss the methods used so far to study the host-viroplasm interactions and emphasize on the potential host factors which can be targeted for therapeutic intervention in the future.

## Introduction

**Figure 1:**
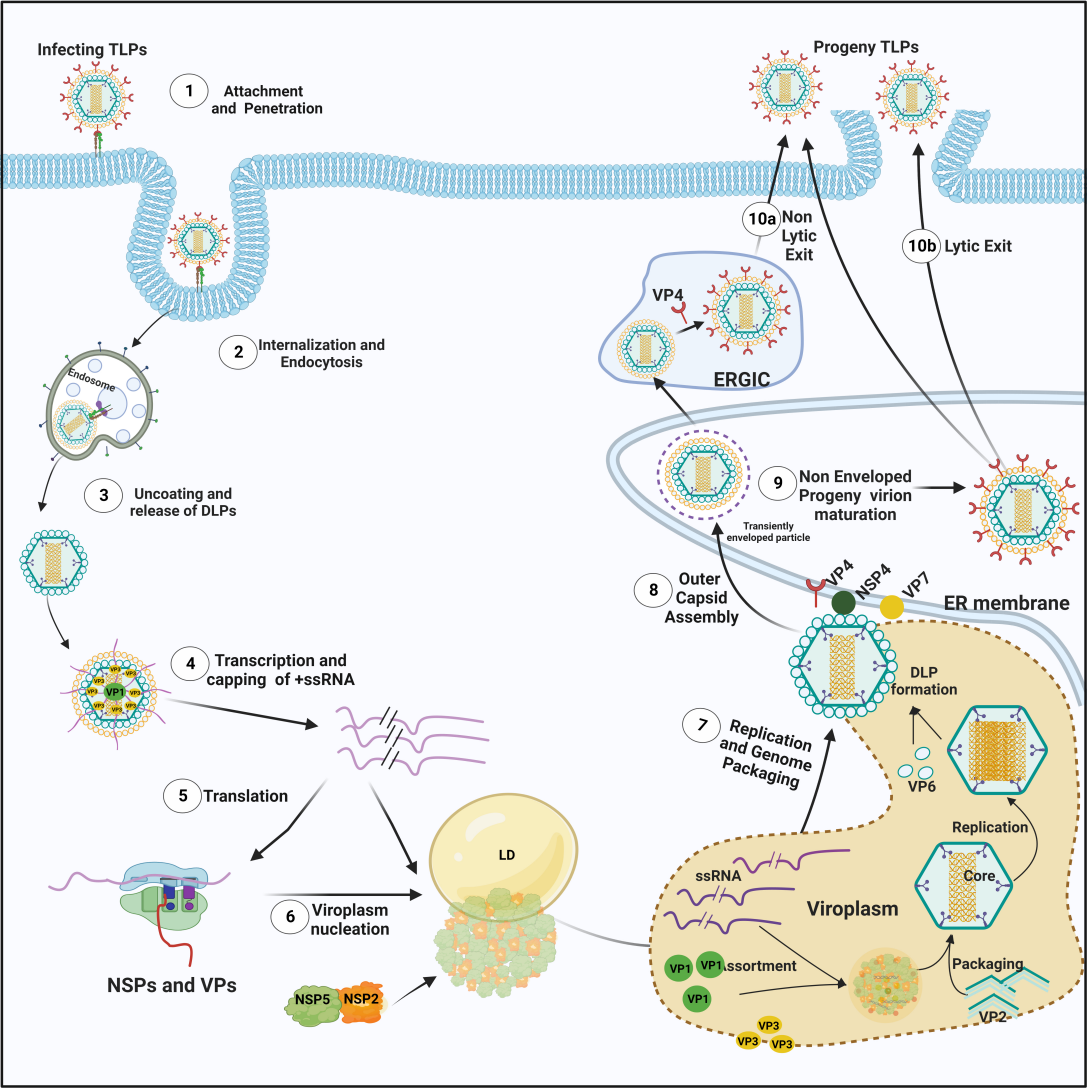
The life cycle of RV. (1) The infectious RV TLPs adhere to the specific receptors present in lipid rafts of the host cellular plasma membrane. (2) Following receptor-mediated endocytosis and trafficking along the endosomal pathway, (3) RV TLPs shed their outermost layer and release DLPs into the cytoplasm. (4) Within DLPs, viral RNAs are transcribed with VP1 acting as a polymerase to yield (+)ssRNAs that are eventually capped by another viral protein VP3. (5) Subsequently, structural and nonstructural proteins are synthesized from RV (+)ssRNAs on cellular ribosomes. (6) NSP5 and NSP2 essentially lead to the nucleation of viroplasms on host cellular lipid droplet (LD) scaffold. (7) Inside the maturing viroplasms, accumulating VP1, VP2 and VP3 participate in the viral genome replication. Within the VP2-encaged viral cores, VP2-driven polymerase activity of VP1 initiates the biogenesis of (-)ssRNAs. De novo synthesized cores acquire the VP6 layer to form the progeny DLPs. (8) The DLPs enter into the morphogenetic assembly pathway by acquiring an outer capsid which occurs by a budding step through the ER-derived cellular membrane where VP6 on DLPs docks on NSP4 on ER-derived membrane. Inside the ER, progeny particles acquire a transient envelope. (9) Subsequently, the transient envelope dissipates, NSP4 is stripped off, and the outermost VP7-VP4 layer is assembled. Alternatively, acquisition of VP4 spikes may occur on VP7-surrounded virions within the ER-Golgi intermediate compartment (ERGIC)/plasma membrane lipid raft domains (10a) before non-lytic virion release. (10b) RV progenies may also exit through lytic mechanisms. Of note, the time kinetics of RV life cycle events is dependent on many factors most important of which are the RV strains and the host cell lines used for infection as well as the multiplicity of infection. In general, in the RV permissive cell line MA104 infected with a simian RV strain SA11 at a multiplicity of infection 3, the timeline of infection is as follows: 0-4 hours post infection (hpi) includes the early life cycle events such as the viral adsorption, entry, endosomal trafficking, initiation of transcription and translation and viroplasm nucleation; 4-8 hpi includes viroplasm maturation and concurrent viral replication, secondary transcription, and initiation of the morphogenetic assembly pathway within maturing viroplasms; 8-12 hpi includes the late life cycle events such as the morphogenetic maturation and viral release.

Rotavirus (RV), the leading cause of viral gastroenteritis among infants and children, primarily infects the enterocytes through fecal-oral transmission route. The subsequent destruction of the absorptive enteric cells and stimulation of the enteric nervous system result in clinical manifestations of RV infection in the form of profuse watery diarrhea, nausea, and vomiting (Crawford et al., 2017). A fully infectious RV is a non-enveloped triple-layered particle (TLP) with three concentric protein layers. The innermost layer contains the structural protein VP2 which ensheathes the 11 segments of rotaviral double-stranded RNA (dsRNA) genome and two other structural proteins VP1 and VP3. The middle layer comprising of trimers of VP6 connects the inner capsid with the outermost layer which is a glycoproteinaceous shell of VP7 with VP4 spikes inserted in it. Both VP4 and VP7 are required for the initial tethering and subsequent attachment of the TLPs to the host cell surface receptors (**Figure 1**) (Silvestri et al., 2004; Lopez and Arias, 2006; Desselberger, 2014). Following attachment, virions are endocytosed, trafficked along the endosomal pathway, and finally released in the host cell cytoplasm as partially unmasked double-layered particles (DLPs) (**Figure 1**) (Lopez and Arias, 2006; Arias and López, 2021). Transcriptionally potent DLPs further enable transcription with the help of VP1 in assistance with VP2 and VP3 to produce capped, non-polyadenylated, positive-sense, single stranded RNAs [(+)ssRNAs] which are subsequently translated into viral proteins [six structural (VP1-4, VP6, VP7) and six non-structural (NSP1-6)] (**Figure 1**). Interestingly, the newly translated non-structural proteins NSP2 and NSP5 mutually interact to form the electron-dense cytoplasmic inclusions bodies, called viroplasms (**Figure 1**). These polyribosome-surrounded viral niches represent the safe house for subsequent viral life cycle events such as viral genome replication, secondary transcription, and early morphogenesis. Although the exact sequence of events is far from clear, VP1 and VP3 along with the transcribed ss(+)RNAs have been shown to form a dynamic association complex where NSP2 joins and acts as an RNA chaperone involved in the pre-genomic RNA assortment. Assembly of decameric VP2 plates around the assorted VP1–VP3–(+)RNA complexes by virtue of VP2’s affinity for VP1, VP3, and ssRNAs forms the innermost core shell. Subsequently, VP2-driven polymerase activity of VP1 initiates the biogenesis of (-)strand RNA (**Figure 1**) (Navarro et al., 2016; Papa et al., 2021). In the early stage, spherical viroplasms have liquid-like properties and are vulnerable to small aliphatic diols which can release nascent transcripts from the condensates (Geiger et al., 2021). The 11 dsRNA genome segments within the replicating progeny cores acquire a peripheral layer of VP6 to form progeny DLPs which can further amplify the replication cycle by producing secondary transcripts or may enter into the morphogenetic assembly pathway (**Figure 1**) (Navarro et al., 2016). The most important morphogenetic event includes the formation of the outermost shell containing VP4 and VP7 on the immature DLPs residing within viroplasms. This requires a budding step through ER-derived cellular membranes where VP6 on DLPs docks on NSP4 on ER-derived membranes along with co-recruitment of VP4 and VP7 on NSP4 (**Figure 1**) (Crawford et al., 2012; Trask et al., 2012; Crawford et al., 2019). The mature TLPs exit ER and finally the infected cells either through lytic mechanisms or by non-lytic secretory pathways which bypass the involvement of Golgi apparatus and lysosomes to continue successive infection cycles (**Figure 1**) (Musalem and Espejo, 1985; Jourdan et al., 1997; Patra et al., 2021).

Core to the fundamental biology of viral life cycle is the obligation to rely on host cellular resources while disarming the host-intrinsic antiviral defense machinery. Not surprisingly, many host components have been reported to be usurped or subverted by RV at different stages of the viral life cycle (Patra et al., 2021). Interestingly, many of such host components are redirected to RV viroplasms and interact with the viroplasmic proteins, raising the notion that RV replication niches not only serve as the safe houses for viral components but also allow selective proximity to certain host factors. Here, we will present a snapshot of various host determinants belonging to the translational and post-translational modification machinery, cytoskeletal elements, lipid droplets, RNA granules, autophagic components, DNA damage, and unfolded protein response which have been observed to associate with viroplasms and regulate their dynamicity at various stages. Interestingly, perturbing many of these host components has been proved to sensitize RV progeny yield, suggesting its important implications in designing host-directed antiviral therapeutics.

## Rotavirus viroplasms and host: An intimate association

RV viroplasms are dynamic structures that form as liquid-like, small, spherical puncta phase-separated within the host cellular cytoplasm. With the progression of infection, these inclusion bodies fuse with each other and grow bigger in size while getting fewer in number and also attain gel-like consistency (Eichwald et al., 2012; Geiger et al., 2021). The essentiality of NSP2 and NSP5 in regulating nucleation of viroplasms has been shown based on two principal lines of arguments - i) co-transfection of NSP5 and NSP2 has resulted in spherical, viroplasm-like structures (VLS) within transfected cells even in the absence other RV proteins (Fabbretti et al., 1999), and ii) loss-of-function of NSP2 and NSP5 severely affects viroplasm formation and viral progeny production (Silvestri et al., 2004; Vascotto et al., 2004; Campagna et al., 2005; Criglar et al., 2018; Papa et al., 2019; Criglar et al., 2020). Notably, apart from NSP2-induced VLS, the formation of VP2-induced VLS has also been reported under the co-transfection scenario of NSP5 with VP2 (Contin et al., 2010).

Importantly, many host cellular components have been reported to associate with RV viroplasms to regulate viroplasm formation and subsequent maturation steps, suggesting the pro-RV significance of these host components. On the other hand, many antiviral host determinants are also sequestered within or around viroplasms whereby they are prevented from exerting their potential antiviral efficacy. In the following sections, we will describe the methods implemented so far to study host-viroplasm interactions and further elaborate on different modalities of these interaction profiles at different stages of the RV life cycle.

## Methods to study host-viroplasm interactions

Staining for the viroplasmic proteins, especially NSP2 and NSP5, with antibodies and subsequent fluorescence imaging under the wide-field or confocal microscope have been the most conventional and go-to-approach for detecting RV viroplasmic puncta. Of interest, based on the affinity for two monoclonal antibodies which are generated against two different conformations of NSP2, two distinct pools of NSP2 were characterized. The dispersed NSP2 (dNSP2) remains evenly distributed in the host cellular cytoplasm whereas the viroplasmic NSP2 (vNSP2) forms distinct puncta and co-localizes with NSP5, corroborating NSP2-NSP5 containing RV inclusion bodies in the host cellular cytosol (Criglar et al., 2014). Not surprisingly, viroplasmic re-localization of many host proteins has been addressed simply by checking the microscopic localization of these proteins with respect to the localization of the viroplasmic RV proteins (Cheung et al., 2010; Zambrano et al., 2011; Criglar et al., 2018; Dhillon and Rao, 2018; Dhillon et al., 2018; Sarkar et al., 2020). Calculating the Pearson correlation coefficient has provided a quantitative assessment of these co-localization events (Dhillon and Rao, 2018; Sarkar et al., 2020). Moreover, the proximity of certain host proteins to viroplasmic proteins has also been assessed by the fluorescence resonance energy transfer (FRET) efficiency (Cheung et al., 2010). Physical interaction between host and RV viroplasmic proteins has been checked by targeted approaches such as doing co-immunoprecipitation (co-IP) coupled to immunoblot studies or through unbiased methods such as affinity-purification coupled to mass spectrometry (AP-MS) (Dhillon and Rao, 2018; Dhillon et al., 2018; Criglar et al., 2020; Sarkar et al., 2020). Sensitivity of the co-IP reactions to RNase A treatment further enabled differentiating the host-viroplasm interactions which are dependent on an intermediate scaffold of RNAs such as the viral RNAs (Dhillon and Rao, 2018; Dhillon et al., 2018). Interestingly, co-localization events observed under a microscope did not always accompany the occurrence of physical interaction identified by AP-MS or co-IP-immunoblot approach, possibly differentiating between proximity-based association and protein-protein interaction (Dhillon and Rao, 2018; Dhillon et al., 2018; Criglar et al., 2020; Sarkar et al., 2020). Indeed, some viral proteins such as NSP2 exist in two different forms. Therefore, assessing the co-localization of host proteins by vNSP2-specific antibody can only confirm the viroplasmic sequestration of host proteins. Moreover, in one particular study, the association between lipid droplets (LDs) and viroplasms has been confirmed by iodixanol density gradient centrifugation where viral dsRNAs and viroplasmic proteins co-sedimented with LD-associated proteins (Gaunt et al., 2013b).

For studying the spatial and temporal dynamicity of viroplasms, viroplasmic proteins were stained and chased over progressive infection time points in infected and fixed cells (Sarkar et al., 2020). Moreover, fusing NSP2 and NSP5 with fluorescent tags such as GFP and mCherry has also been used to study viroplasm dynamics through time-lapse video microscopy. Making stable cell lines for these wild type and different mutant reporter constructs in a trans-complementing setting where the infection is established by recombinant RV (rRV) deficient in the corresponding gene(s) has provided an ideal way to address the functionality of these viral proteins (Eichwald et al., 2012; Papa et al., 2019; Jing et al., 2021).

## Host cellular contribution in the early stages of viroplasm formation

**Figure 2:**
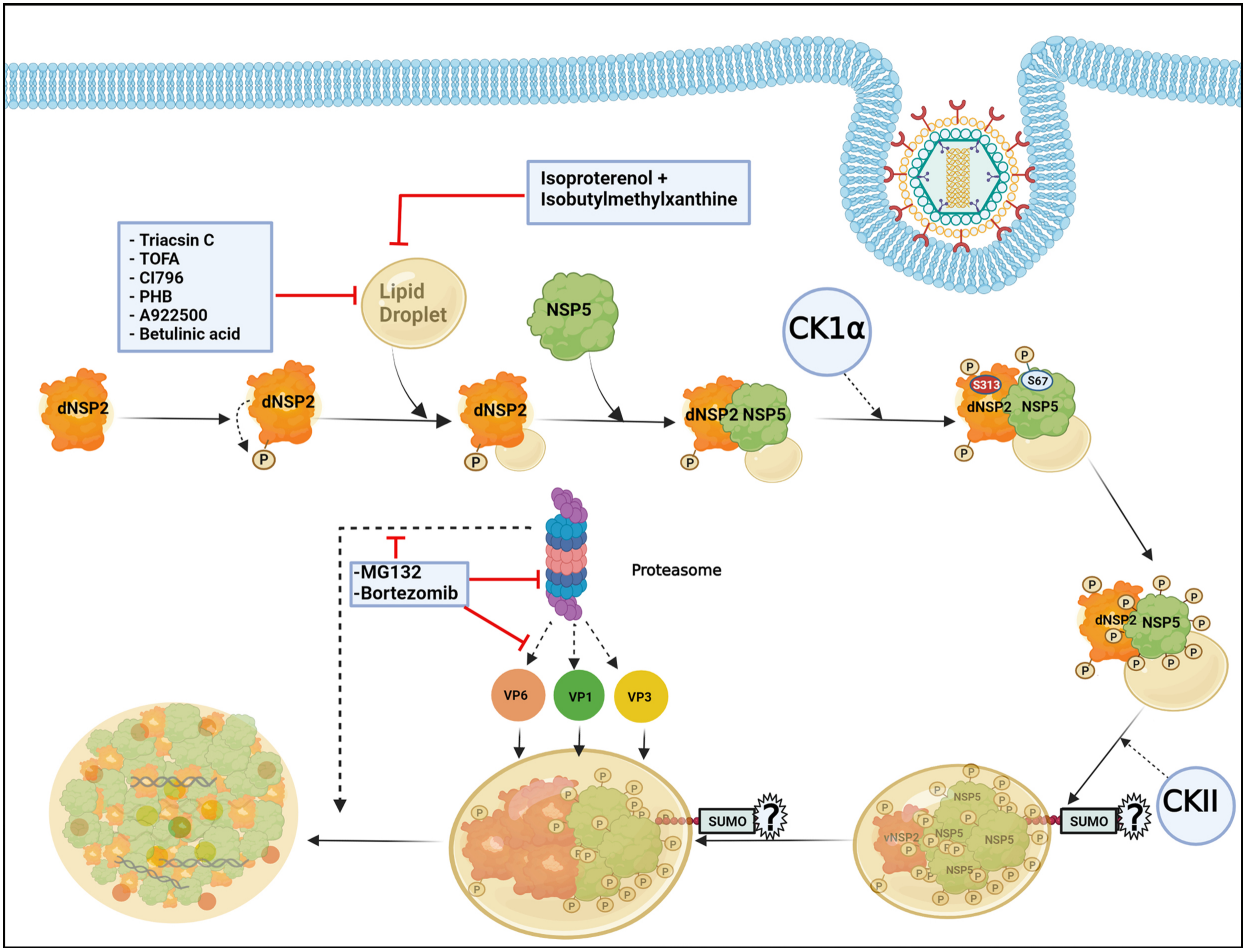
Host cellular contribution in regulating early viroplasm dynamics. Following entry to the host cell, RV NSP2, and NSP5 initiate viroplasm biogenesis. Nucleation of the viroplasm starts with the cytoplasmic, dispersed NSP2 (dNSP2) which phosphorylates itself by the intrinsic NTPase and autokinase activity. NSP2 further interacts with Lipid droplets (LDs) and hypophosphorylated NSP5. Host cellular protein CK1α localizes to viroplasms and phosphorylates NSP2 (at serine 313) and NSP5 (at serine 67). NSP5 is also phosphorylated by another host cellular kinase CKII and by the kinase activity of NSP2. Gradually, NSP5 becomes hyperphosphorylated and dNSP2 gets converted into viroplasmic NSP2 (vNSP2) through conformational changes. The size of the viroplasms and LDs continues to increase over time and the maturing viroplasms accumulate other viral proteins such as VP1, VP2, and VP6. An active ubiquitin proteasome system (UPS) is required for the early dynamics of viroplasms. Proteasome inhibition by small molecules (by MG132 and Bortezomib) leads to smaller viroplasmic size and abrogated translocation of VP1, VP2, and VP6 into the viroplasms. Inhibiting LD biogenesis by targeting the enzymes of the LD synthesis pathway or promoting LD fragmentation compromises early viroplasm dynamics. Notably, SUMOylation of NSP5 at multiple lysine residues has been shown to be important for NSP5-VP2 induced viroplasm-like structure (VLS) formation. However, the importance of NSP5 SUMOylation in viroplasm formation under natural infection scenarios is questionable.

The formation of viroplasms requires not only the viroplasmic proteins NSP2 and NSP5 but also their phosphorylated forms which are primarily generated by the cellular kinases casein kinase 1α (CK1α) and casein kinase II (CKII) (Eichwald et al., 2004; Campagna et al., 2007; Criglar et al., 2018; Papa et al., 2019). Viroplasm nucleation starts with the dNSP2 which gets autophosphorylated by its intrinsic NTPase and autokinase activity and further associates with the hypophosphorylated NSP5 (26 kDa) (**Figure 2**). Further, CK1α co-localizes with the dNSP2-NSP5 complex and phosphorylates dNSP2 at Serine 313 (S313) residue (**Figure 2**). NSP5 gradually attains its hyperphosphorylated state including a priming phosphorylation at Serine 67 (S67) residue by the continued kinase activity of CK1α and/or NSP2 (**Figure 2**). Concurrently, the dNSP2–NSP5 complex traffics to the putative viroplasm nucleation sites, associates with LDs, and forms the vNSP2/hyperphosphorylated NSP5 complex within maturing viroplasms (Criglar et al., 2018; Papa et al., 2019). Interestingly, silencing the expression of CK1α drastically affected dNSP2-to-vNSP2 conversion and NSP5 hyperphosphorylation, leading to compromised viroplasm formation and RV progeny production (Criglar et al., 2018). Moreover, a rRV with S67A NSP5 mutation also fails to trigger NSP5 hyperphosphorylation, and proves to be defective in viroplasm assembly and infectious progeny yield (Papa et al., 2019). On a consistent note, a phosphomimetic NSP2 mutant (S313D) shows delayed kinetics of viroplasm assembly (Criglar et al., 2020), corroborating host directed phosphorylation events on NSP2 and NSP5 to have crucial regulatory roles on RV viroplasm formation. In addition to CK1α, implications of CKII-mediated phosphorylation events have also been suggested for NSP5 to form higher order oligomeric complex that exists in mature viroplasms (**Figure 2**) (Papa et al., 2019).

In addition to phosphorylation, NSP5 has been reported to get modified by small ubiquitin-like modifier (SUMO) molecules at multiple lysine (K) residues, suggesting that the host cellular SUMO system might potentially influence the architecture and functionality of RV viroplasms (**Figure 2**) (Campagna et al., 2013). The exact nature of SUMO conjugation on NSP5, especially whether NSP5 undergoes multi-mono SUMOylation (single SUMO moiety attached to multiple K residues) or polySUMOylation (internally linked SUMO chain attached to a single K residue) or both, is not confirmed. A possible correlation between NSP5 phosphorylation and SUMO conjugation has been hypothesized based on the observation that the in vitro translated NSP5 SUMO mutant shows increased phosphorylation. Moreover, the mutant NSP5 fails to form VLS when co-expressed with VP2, suggesting the importance of NSP5 SUMO modification to potentially influence its interaction with VP2. On the contrary, this mutant retains its ability to interact with NSP2 as well as VP1, and forms VLS when co-expressed with NSP2. Ectopic expression of this SUMO mutant is also as potent as the wild type NSP5 in restoring infection in NSP5-depleted infected cells. Moreover, neither overexpression of SUMO isoforms or silencing of Ubc9 (one of the enzymes in the SUMO conjugation pathway) affects NSP5 phosphorylation nor SUMOylation of NSP5 gets perturbed in presence of co-expressing NSP2, VP2, or VP1, arguing against a probable correlation between NSP5 SUMOylation and phosphorylation (Campagna et al., 2013). Therefore, the question remains regarding the significance of host cellular SUMO system and SUMO conjugation in regulating RV viroplasm formation during natural infection scenario when NSP2-NSP5 interaction initiates viroplasm nucleation and SUMO isoforms are not overexpressed. Moreover, the SUMO system is mostly nuclear in localization whereas RV viroplasms are exclusively cytosolic, raising the question of where the intersection occurs in infected cells. Of interest, overexpression of SUMO isoforms and depletion of Ubc9 antagonize RV infection as a whole, indicating potential implications of SUMOylation on other parts of RV life-cycle. Indeed, other viroplasmic components (VP1, VP2, VP6, NSP2) can be covalently SUMO conjugated or can interact with SUMO in a noncovalent manner (VP1, VP2, and NSP2) (Campagna et al., 2013).

LDs are the intracellular lipid storage organelles involved in different cellular phenomena including lipid homeostasis, signal transduction, and membrane trafficking (Ohsaki et al., 2014; Crawford and Desselberger, 2016). Structurally, LDs consist of a core of triacylglycerols (TAG) and sterol which is surrounded by a phospholipid monolayer where LD-associated proteins such as adipose differentiation-related protein (ADRP) and perilipins are inserted (Kimmel et al., 2010). LDs have been shown to be usurped by RV to foster viroplasm formation during the early hours of infection in cell culture and also in the human intestinal organoid model (Cheung et al., 2010; Foulke-Abel et al., 2014; Saxena et al., 2015). Despite mechanistic details of how viroplasms interact with the LDs are lacking, conformational changes in NSP2 or NSP5 during viroplasm nucleation have been postulated to expose lipophilic residues (NSP5 possesses an amphipathic helix) of the proteins which might further be inserted into the LD membranes, thereby assembling an amphipathic complex with LDs (Criglar et al., 2018). Agreeably, stains for viroplasmic proteins merge with different lipophilic stains and co-localize with LD-associated proteins (perilipin A, phospho-perilipin, and ADRP) in infected cells (Cheung et al., 2010; Criglar et al., 2020; Criglar et al., 2022). Moreover, both perilipin1 and phospho-perilipin1 co-immunoprecipitate vNSP2, suggesting a possible interaction between NSP2 and LD proteins (Criglar et al., 2020). Interestingly, LDs were also observed to be associated with VLS formed by NSP2 and NSP5, indicating their involvement in promoting viroplasm assembly irrespective of the RV replication potency (Cheung et al., 2010). In fact, taking advantage of the delayed viroplasm dynamicity in cells infected with the rRV which has the phosphomimetic (S313D) NSP2, LD-NSP2 association was found to precede NSP2-NSP5 interaction (**Figure 2**) (Criglar et al., 2020). As the infection progresses, the size of both the viroplasms and LDs increase (Eichwald et al., 2012), indicating that viroplasms assemble concomitantly with LD biogenesis (**Figure 2**). Consequently, when RV-infected cell extracts (detergent-free) were subjected to equilibrium ultracentrifugation through iodixanol gradients, viral dsRNAs were co-sedimented with NSP5, perilipin A, and lipids that reside in LDs in the same low-density fraction, further corroborating a lasting association of LDs with RV viroplasms (Cheung et al., 2010; Gaunt et al., 2013b). In agreement with the pro-rotaviral significance of cellular LDs in regulating viroplasms, interrupting LD homeostasis with small molecules heavily antagonized viroplasm formation and viral progeny production. Several enzymes belonging to the neutral lipid biosynthetic pathway [such as long-chain acyl-CoA synthetase (ACSL), acetyl-CoA carboxylase 1 (ACC-1), fatty acid synthase (FASN) complex, diacylglycerol acyltransferases (DGAT1, DGAT2), acyl-coenzyme A (CoA):cholesterol acyltransferases (ACAT1 and ACAT2)] have been proved to be anti-RV targets for intervention by small molecules [ACSL by triacsin C, ACC-1 by 5-(tetradecyloxy)-2-furoic acid (TOFA), FASN by C75, DGAT by A922500 or betulinic acid, and ACAT by CI-976 or PHB] (**Figure 2**). Notably, TOFA also interfered with the assembly step of RV outer capsid, showing drug synergism with C75 (Cheung et al., 2010; Kim et al., 2012; Gaunt et al., 2013a; Crawford and Desselberger, 2016). Moreover, LD fragmentation (by a combination of isoproterenol + isobutylmethylxanthine) antagonized RV replication and RV-induced cytopathy (**Figure 2**) (Cheung et al., 2010).

Among the other host determinants which contribute to the early viroplasm dynamics, implications of ubiquitin proteasome system (UPS) have been cited (**Figure 2**). UPS enables the turnover of proteins via proteolytic ubiquitylation coupled to proteasomal degradation and therefore is an important component of cellular proteostasis. A functional UPS is required for effective RV replication as proteasome inhibition significantly reduced viral protein and RNA levels as well as viral progeny yield (Contin et al., 2011; López et al., 2011). Mechanistically, proteasome inhibition by small molecules (such as MG132 and Bortezomib) and RNA interference (RNAi) (targeting components of the UPS) heavily sensitized the formation of viroplasms (**Figure 2**). A time-of-addition study showed that treatment with proteasome inhibitors resulted in stunted viroplasm assembly in the form of smaller viroplasms, suggesting the importance of functional proteasome in regulating early viroplasm dynamics (Contin et al., 2011). Moreover, proteasome inhibition also led to the failure of VP1, VP2, and VP6 to be effectively incorporated into the poorly formed viroplasmic puncta (**Figure 2**), justifying compromised genome replication and progeny yield of RV (López et al., 2011). Of interest, sensitivity to UPS inhibition was only evident for viroplasms formed during natural infection, but not for VLS which are formed upon co-expression of NSP5 with NSP2 or VP2. Similarly, overexpressed VP1 and VP6 did not fail to co-localize with VLS in presence of proteasome inhibitors, suggesting the relevance of UPS for actively replicating RV with dynamic viroplasmic architecture (Contin et al., 2011).

## Host cellular contribution in viroplasm maturation and dynamicity

**Figure 3:**
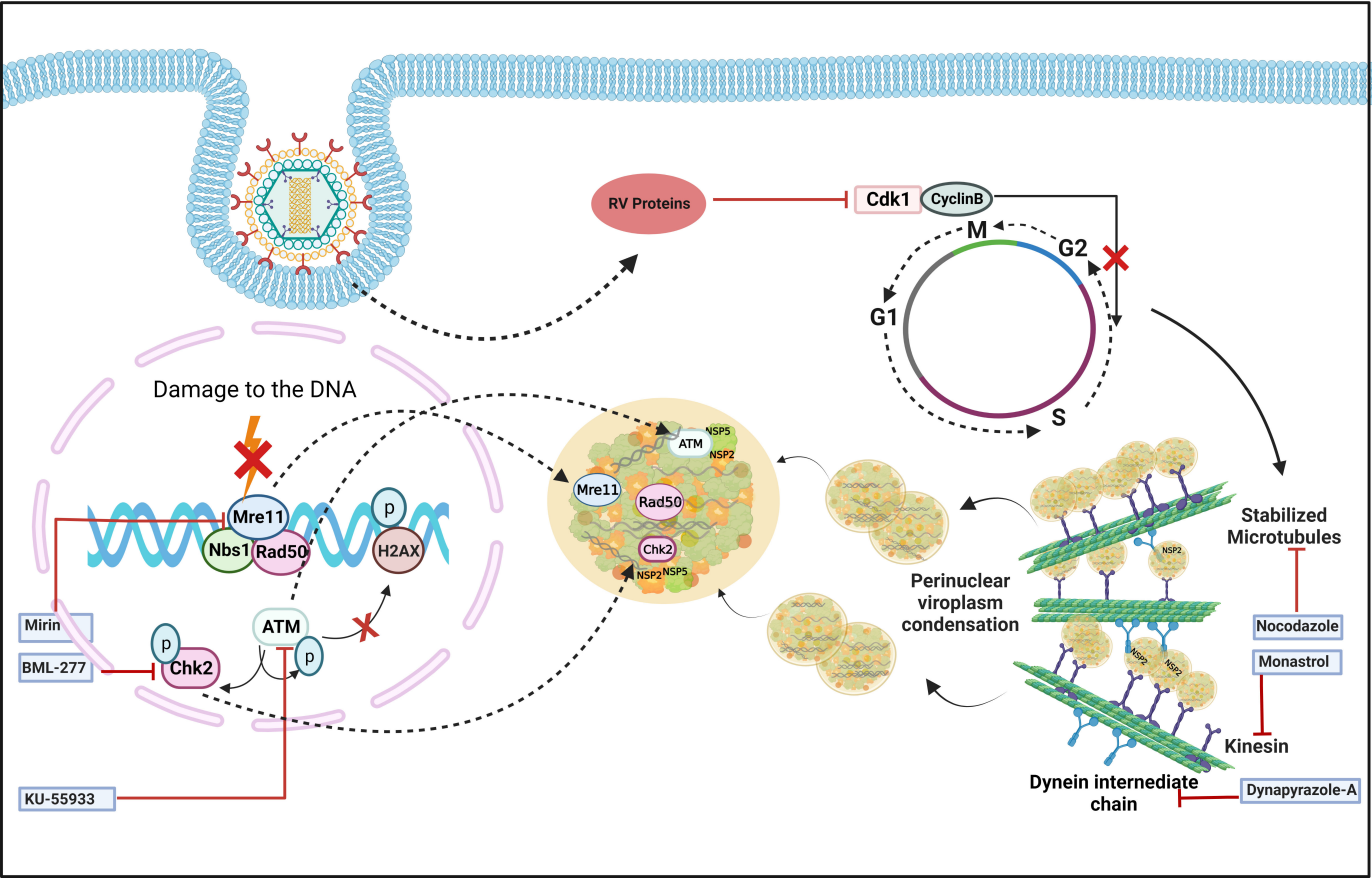
Host cellular contribution in regulating viroplasm condensation and maturation. RV blocks mitotic entry of the host cell cycle by prolonging intra S-phase retention. This is enabled by the depletion of cyclin B1 and subsequent inhibition of the Cdk1-cyclin B1 complex by multiple RV proteins. Inhibition of mitotic entry ensures the preservation of hyperacetylated and stabilized microtubular structures which along with the kinesin motor protein Eg5 and dynein facilitate viroplasmic condensation and peri-nuclear relocalization. Targeting microtubule, Eg5 kinesin, and dynein intermediate chain by small molecules nocodazole, monastrol, and dynapyrazole-A respectively, impairs viroplasm dynamics. RV also activates MRN-ATM-Chk2 branch of DDR during infection in absence of nuclear DNA damage and λ-H2AX positive nuclear foci. Moreover, MRN components, ATM and Chk2 relocate to the cytoplasm and co-localize with viroplasms. Targeting Mre11, ATM, and Chk2 by small molecule inhibitors Mirin, KU55933, and BML-277, respectively, antagonize RV replication. KU55933 and BML-277 prevent viroplasm condensation and maturation.

In addition to viroplasm nucleation, growth and maturation of these viral structures also require host cellular assistance. Confocal microscopy showed that viroplasms are dynamic structures where small nucleating puncta fuse with each other to form smaller number of bigger aggregates which subsequently gather in the perinuclear space during the course of infection (**Figure 3**) (Eichwald et al., 2012). Importantly, to achieve such dynamicity, RV usurps the host microtubular network which consists of heterodimers of α- and β-tubulin, and regulates the intracellular transport of organelles and macromolecules. Two types of molecular motors, dyneins, and kinesins are the chief mediators of this microtubule-mediated intracellular transport. RV NSP2, but not NSP5, interacts with tubulin dimers via its positively charged grooves to sequester tubulins inside viroplasms, resulting in a sharp decrease in cytoplasmic tubulin concentration and co-localization of viroplasms with microtubule granules (Martin et al., 2010). As a result, microtubule depolymerization takes place, possibly disrupting the host cellular trafficking processes. Counterintuitively, actively replicating RV has been shown to trigger prolonged intra-S phase retention of the host cells (Glück et al., 2017) and utilizes the stabilized microtubular structures for maintenance and maturation of the viroplasms (**Figure 3**). Stabilization of microtubules was enabled by the increased acetylation of tubulin which embeds the viroplasms. Microtubule depolymerizing drug Nocodazole blocks viroplasm growth and peri-nuclear fusion without hampering the nucleation step (**Figure 3**) (Eichwald et al., 2012). In addition to microtubules, viroplasm maturation and perinuclear re-localization are also governed by kinesin protein of the Eg5 family and are therefore sensitive to an allosteric inhibitor of the Eg5 kinesin, monastrol (**Figure 3**) (Eichwald et al., 2012). A recent report has also indicated the requirement of a microtubule-associated dynein transport system for rotaviral propagation (Jing et al., 2021). The dynein transport apparatus is associated with viroplasm formation at both early and late stages of RV life cycle. RV viroplasms are found to co-localize with the dynein intermediate chain (DIC) and physical interaction was also evidenced between RV NSP2 and DIC (**Figure 3**). Viroplasms exploit dynein to avail the retrograde transport to move along the microtubules. This subsequently facilitates the fusion of two small viroplasms into a bigger one, which further promotes viral progeny replication. DIC inhibition by RNAi and by small molecules such as dynapyrazole-A attenuated both the size and number of viroplasms, leading to curtailed viral progeny synthesis (**Figure 3**) (Jing et al., 2021).

The dynamicity of RV viroplasms has recently been shown to be influenced by the host cellular DNA damage response (DDR) factors. DDR is primarily a nuclear phenomenon where sensing of damage on the DNA by a group of proteins called sensors is followed by transducing the damage signal via another group of proteins named transducers to the effectors which either repair the damage while arresting the cell cycle or induce cellular demise by apoptosis (Bartek and Lukas, 2001; Luftig, 2014). Actively replicating RV was found to trigger activation of the damage sensor complex Mre11-Rad50-Nbs1 (MRN) followed by stimulation of the transducer kinase ATM and its downstream effector Chk2 (**Figure 3**) (Sarkar et al., 2020). Interestingly, induction of the MRN-ATM-Chk2 pathway during RV infection neither depends on the occurrence of nuclear DNA damage nor leads to the formation of damage-induced canonical nuclear foci (**Figure 3**), indicating a non-canonical response in the infected cells. Moreover, components of the MRN complex as well as ATM and Chk2 were reported to get relocated from nucleus to cytoplasm and to co-localize with RV viroplasms (**Figure 3**). ATM and Chk2 were also found to interact with the viroplasmic RV proteins NSP2 and NSP5 under infection scenario. Inhibiting Mre11, ATM, and Chk2 by small molecules Mirin, KU55933, and BML-277, respectively, antagonized RV progeny yield. Chasing viroplasms with ATM and Chk2 inhibitors in a time-of-addition study revealed that the ATM-Chk2 pathway is important for the fusion and maturation of viroplasms and subsequent viral propagation (**Figure 3**). Agreeably, nucleation of viroplasmic puncta and disintegration of already formed viroplasms were not sensitized by inhibition of this pathway. Of interest, co-expressing NSP2 and NSP5 could mimic neither the activation of the MRN-ATM-Chk2 pathway nor the cytosolic relocation and viroplasmic sequestration of MRN components, ATM, and Chk2 (Sarkar et al., 2020). The molecular rationale behind usurping a branch of DDR in a skewed, non-canonical way in favour of facilitated viroplasm fusion and productive viral perpetuation is still an open question.

## Sequestering host components within viroplasms to prevent their canonical functions

**Figure 4:**
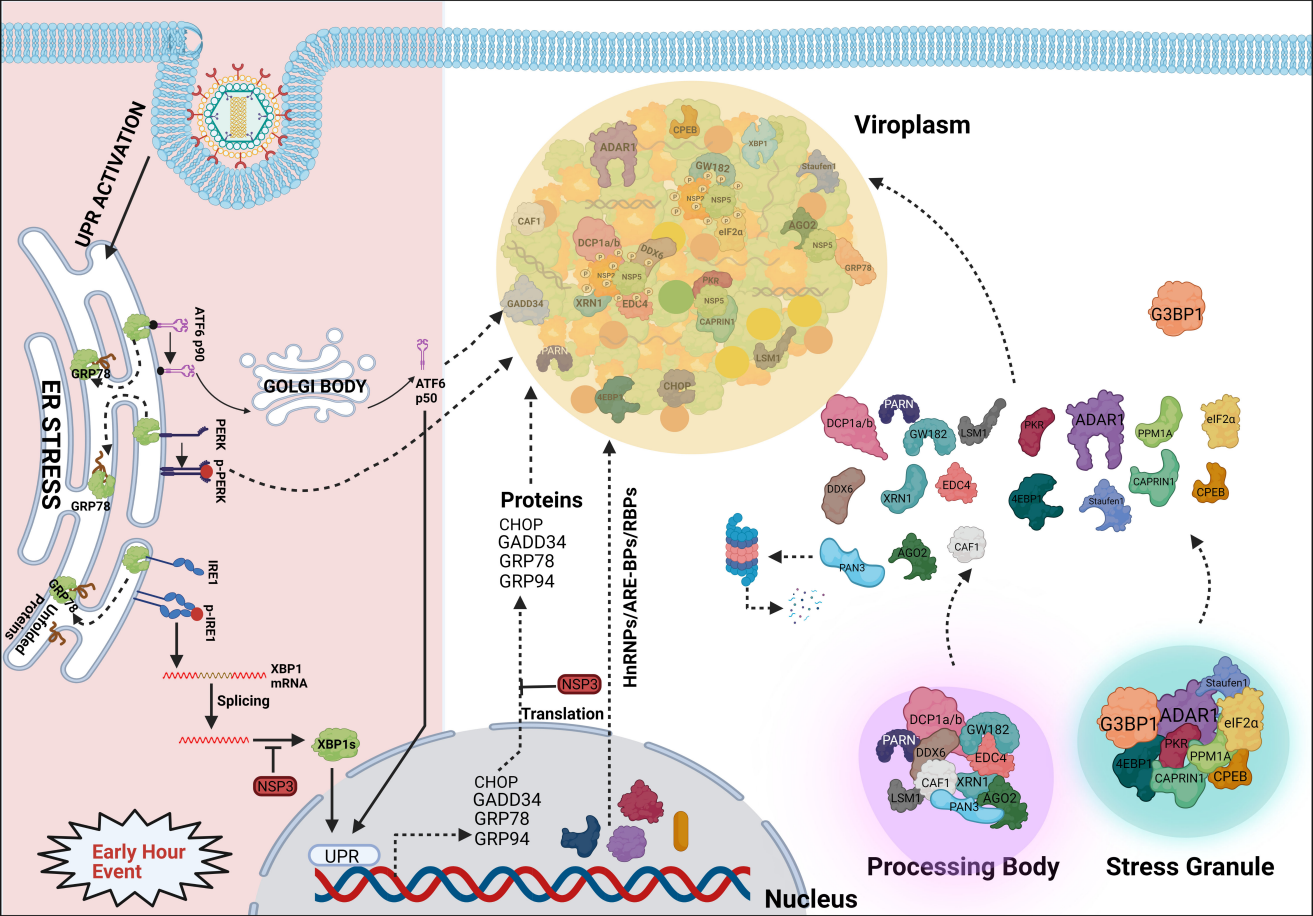
Sequestration of different host components into viroplasmic condensates. RV dismantles P bodies and SGs during infection. Many of the SG (ADAR1, eIF2α, Caprin1, PKR, Staufen1, CPEB, 4EBP) and P body (DDX6, Lsm1, Caf1, PARN, XRN1, DCP1a, DCP1b) components relocate to viroplasms at least transiently or partially and interact with viroplasmic proteins. Additionally, many hnRNPs and ARE-BPs are re-located from the nucleus to the cytosol, get sequestered within viroplasms, and interact with NSP2 and NSP5 within RV infected cells. Many relocated RBPs are also absorbed by the viral transcripts. Accumulation of misfolded proteins in the ER leads to uncoupling of GRP78 from UPR sensors, resulting in activation of the three branches of UPR-the ATF6 pathway, the PERK-dependent pathway, and the IRE1-based signalling. During early hours of infection, RV activates the ATF6 and IRE1 branches of the UPR. Following dissociation from GRP78, the transcriptionally inactive fragment of ATF6 (ATF6p90) is translocated to the Golgi apparatus where it is cleaved into the transcriptionally active fragment (ATF6p50) which is further transported to the nucleus to trans-activate UPR elements (CHOP, GADD34, GRP78 and GRP94). Despite initial activation of the ATF6 branch of UPR, RV inhibits transcription of UPR elements by sequestering the ATF6p50 fragment into viroplasms. Moreover, activation of the IRE1 pathway includes dimerized and autophosphorylated IRE1 (p-IRE1) to trigger splicing of xbp1 mRNA (xbp1u) to generate a spliced variant (xbp1s). However, further translation of the xbp1s is prevented as a result of the host translational inhibition mediated by RV-NSP3. Release of PERK from GRP78 leads to homo-dimerization and phosphorylation of PERK; however, RV sequesters p-PERK in the viroplasms inhibiting further activation. Additionally, RV blocks the potential antiviral effects of UPR by inhibiting the translation of ER stress responsive genes and by re-locating many UPR effector proteins (CHOP, GADD34, GRP78, GRP94) near/within viroplasms at the late hours of infection.

RV viroplasms have recently been shown to form as phase-separated macromolecular condensates within the host cellular cytoplasm (Geiger et al., 2021). Interestingly, eukaryotic cells also possess membrane-less organelles such as processing bodies (P bodies) and stress granules (SGs) which represent phase-separated cytoplasmic condensates. These dynamic mRNA-protein inclusion foci are involved in the cellular RNA surveillance. Partitioning of eukaryotic mRNA between polysomes, SGs, and PBs/GW-bodies has been reported to dictate the fate of mRNA population by governing the rate of mRNA translation and mRNA repression/degradation/decay which further regulate gene expression (Reineke and Lloyd, 2013; Riggs et al., 2020). RV infection triggers host cellular translational arrest which may lead to activated host RNA surveillance that might have potentially deleterious effects on unrestricted translation of viral mRNAs on cellular polysomes. Interestingly, RV has been shown to prevent the formation of canonical P body and SG condensates (**Figure 4**) (Montero et al., 2008; Bhowmick et al., 2015; Green and Pelkmans, 2016; Dhillon and Rao, 2018; Oceguera et al., 2018). Mechanistically, this is enabled by the degradation of selective PB/SG components and by the re-organization of the granular components to different subcellular locations (**Figure 4**) (Montero et al., 2008; Bhowmick et al., 2015; Dhillon and Rao, 2018; Oceguera et al., 2018). Many P body (DDX6, Lsm1, Caf1, PARN, XRN1, DCP1a, DCP1b), GW body (AGO2), and SG proteins (ADAR1, CPEB, eIF2α, 4EBP1, PKR, Staufen1) have been shown to get re-located to RV viroplasmic puncta, thereby constituting a “molecular triage” (**Figure 4**). Moreover, some of these components also interact with the viroplasmic proteins NSP2, NSP5, and VP6 either via an intermediate RNA scaffold or independent of the viral RNAs (**Figure 4**) (Dhillon and Rao, 2018). The significance of viroplasmic sequestration of each of these individual proteins during RV infection awaits further mechanistic studies. Of importance, some of these relocated proteins such as the decapping complex components DDX6, XRN1, DCP1a, DCP1b showed partial and transient association with viroplasms (Bhowmick et al., 2015; Dhillon and Rao, 2018). The most notable example is AGO2 which undergoes degradation during the early hours of infection but is rescued later on and relocates to the viroplasmic niche (**Figure 4**) (Dhillon and Rao, 2018; Mukhopadhyay et al., 2019b). It is interesting to mention here that some of the re-located cytosolic mRNA surveillance proteins such as PKR, Staufen1, and ADAR1 have dsRNA binding domains (Saunders and Barber, 2003). ADAR1 has the property of introducing hypermutation within mRNA (RNA editing) and/or suppression of PKR. During RV infection, the presence of dsRNA, possibly of viral origin, has been detected beyond the viroplasmic confinement in the host cellular cytosol (Rojas et al., 2010; Zhu et al., 2017), and dsRNA-dependent PKR activation has been shown to contribute to eukaryotic initiation factor 2α (eIF2α) phosphorylation, leading to host translational repression (Rojas et al., 2010). Therefore, confining ADAR1 near viroplasms might facilitate PKR activation by the cytoplasmic dsRNA pool; however, viroplasmic sequestration of PKR and eIF2α has also been evidenced at late hours of infection (Dhillon and Rao, 2018). More direct involvement of ADAR1 in fostering RV replication becomes evident when depleting ADAR1 reduces RV progeny yield and overexpressing ADAR1 elevates RV titre (Dhillon and Rao, 2018). Other cytosolic RNA quality control pathways such as the Staufen mediated mRNA decay (SMD) and non-sense mediated mRNA decay (NMD) are also averted by RV, possibly by disarming Staufen-1 within viroplasmic confinement and degrading UPF1, the chief effector RNA helicase involved in both SMD and NMD, by the viroplasmic protein NSP5 (Dhillon and Rao, 2018; Sarkar et al., 2022). Unlike ADAR1, RNAi-mediated silencing of many SG/P body proteins resulted in increased viroplasmic protein (NSP2, NSP5, VP6) expressions and enhanced infectious progeny yield, indicating the antiviral importance of these host cellular determinants. Consistently, ectopic overexpression of some of these potentially antiviral host proteins (G3BP1, Caprin, Dcp1a, Caf1) resulted in reduced rotaviral titer (Dhillon and Rao, 2018).

Several heterogeneous nuclear ribonucleoproteins (hnRNPs) such as hnRNP C1/C2, D, E, F/H, I, K/J, L, and U have been reported to undergo cytosolic relocalization and viroplasmic sequestration in RV infected cells (**Figure 4**) (Dhillon et al., 2018). Moreover, these relocated hnRNPs have been found to interact with the viroplasmic RV proteins NSP2 and NSP5 in absence of any intermediate RNA scaffold (**Figure 4**). Interestingly, similar to the relocated DDR components, these translocation events were not observed in NSP2-NSP5 co-transfected cells, suggesting the involvement of active viral replication and other RV proteins. The significance of such relocation, however, is far from clear (Dhillon et al., 2018). Given that hnRNPs represent a huge family of RNA-binding proteins (RBPs), one hypothesis might be that this relocation and sequestration are stochastic. According to this view, all these RBPs are inherently nucleocytoplasmic shuttling proteins and can be recruited to the RV RNAs because of a general “sponging” effect (Oceguera et al., 2018). Indeed, RV mRNAs present 57 to 68% A + U content with UU, UA, and AU sequences being uniformly distributed along the mRNA length, suggesting the possibility of absorbing re-located AU-rich element binding proteins (ARE-BPs) (**Figure 4**) (Dhillon et al., 2018). On a consistent note, the nucleus-to-cytosolic shuttling of some RBPs was found to be sensitive to viral RNA depletion (Oceguera et al., 2018). hnRNPs and ARE-BPs are involved in numerous aspects of nucleic acid metabolism including mRNA stabilization, alternative splicing, transportation of mRNAs from the nucleus to the cytoplasm, transcriptional and translational regulation, and maturation of the pre-mRNA (Geuens et al., 2016), making them a target for usurpation or subversion by many viruses (Valente and Goff, 2006; Rozovics et al., 2012; Cathcart et al., 2013; Pingale et al., 2020). However, direct implications of host cellular RBPs in specifically modulating RV genome replication, transcription, and translation are yet to be addressed. Nonetheless, significance from the viral perspective is evident as RNAi-mediated silencing and plasmid-based overexpression of HuR, hnRNP D, hnRNP I, and hnRNP K led to diminished and increased progeny virus production, respectively. Other components (G3BP1, TIA1, and hnRNP C1) showed antiviral potency as their down-regulation facilitated RV infection and ectopic overexpression antagonized progeny virus yield (Dhillon et al., 2018). Moreover, for some of the hnRNPs, overexpression or silencing only sensitized modulation of selective RV viroplasmic protein levels, suggesting the possibility that hnRNPs might be exploited by RV in a highly selective manner such as regulating translation of specific viral mRNAs (Dhillon et al., 2018).

In addition to RBPs and hnRNPs, many effector proteins of the unfolded protein response (UPR) pathway such as PKR-like ER kinase (PERK), C/EBP homologous protein (CHOP), Growth arrest and DNA damage-inducible protein (GADD34), activating transcription factor 4 (ATF4), and ATF6 have been observed to get re-localized to or near viroplasms at late hours of RV infection (**Figure 4**) (Zambrano et al., 2011). In RV infected cells, host translation is stagnated partially because of PKR-mediated eIF2α phosphorylation and evidence of activated UPR has been found (**Figure 4**) (Montero et al., 2008; Rojas et al., 2010; Trujillo-Alonso et al., 2011). UPR effectively allows cellular protein homeostasis by preventing the stress-induced accumulation of misfolded proteins within ER. This is enabled by reducing the global translation rate and upregulating the synthesis of selective, stress-responsive transcription factors which further trans-activate UPR-responsive genes such as chaperones or pro-apoptotic factors (Hetz and Papa, 2018). Acutely replicating virus might inadvertently trigger host cellular ER stress leading to activation of the UPR which may heavily antagonize viral replication (Mehrbod et al., 2019). Indeed, the antiviral importance of CHOP as a pro-apoptotic factor has been evidenced (Medigeshi et al., 2007; Kim et al., 2009; Turpin et al., 2021). Moreover, GADD34 has been shown to recruit protein phosphatase 1 (PP1) leading to PP1-mediated dephosphorylation of eIF2α which initiates host cellular translation (Dalet et al., 2017). Therefore, viroplasmic sequestration of these UPR effectors might be an evasive strategy adopted by RV to avoid the potentially deleterious effects of UPR-dependent antiviral host responses. Of note, viroplasmic sequestration of UPR proteins comes as a secondary safeguard against UPR as the primary defense mechanism includes a translational block of these UPR transcripts by the RV non-structural protein NSP3 (**Figure 4**) (Trujillo-Alonso et al., 2011).

Several reports from independent research groups have now demonstrated that host cellular proteins with canonical nuclear annotations and functionality undergo cytosolic re-localization and sequestration within or around viroplasms, suggesting the likelihood of dysregulated nucleocytoplasmic transport during RV infection (Dhillon and Rao, 2018; Dhillon et al., 2018; Sarkar et al., 2020). This dysregulation is not a stochastic phenomenon as DAPI staining ensured nuclear integrity and the translocation events are found to be specific but not a non-selective exodus. Interestingly, changes in the sub-cellular levels of nucleocytoplasmic transport factors have also been observed in RV-infected cells as infection triggered time point-dependent increase of Exportin1, Importin-β, Ran in cytosolic fractions and reduction of Transportin1 in nuclear fractions. Moreover, all these shuttling factors were shown to co-localize with viroplasms and co-IP studies confirmed interactions between Exportin1 and NSP5 as well as between NSP2 and importin-β, Ran (Dhillon et al., 2018). However, whether these nucleocytoplasmic transport factors are involved in directly regulating sub-cellular partitioning of proteins in RV-infected cells is yet to be addressed.

## Host cellular contribution in the late stages of viroplasm dynamics

**Figure 5:**
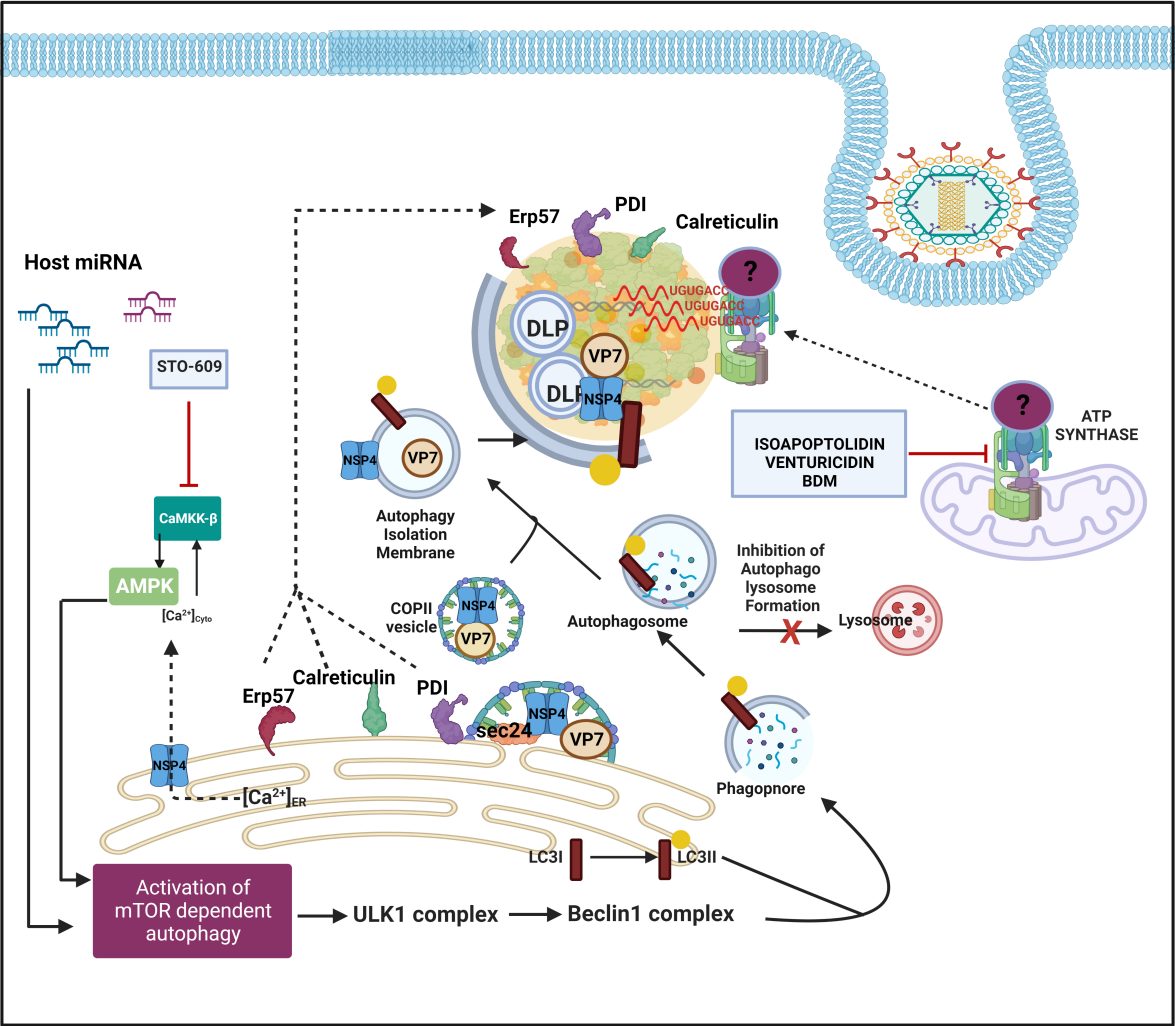
Host cellular contribution in the later events of viroplasm dynamics. Many ER chaperone proteins such as Erp57, PDI, and Calreticulin translocate near RV viroplasms. PDI and Calreticulin foster RV morphogenesis. In RV infected cells, mTOR inhibition and the autophagic signalling are induced by host microRNA-dependent mechanism and also via NSP4-Ca2+-Calmodulin-CaMKKβ-AMPK pathway. Overall, mTOR restriction causes de-repression of the ULK1 complex which subsequently forms phagophore through Beclin1 complex activation and LC3 II lipidation. However, autophagosomes are prevented from lysosomal targeting in RV infected cells; instead, they are utilized for carrying the RV proteins NSP4 and VP7 coming out with the ER-derived COP-II vesicles to maturing progeny virions within viroplasms, thereby aiding in outer capsid assembly. Inhibiting CaMKKβ by STO-609 abrogates the presence of NSP4 and VP7 to reside surrounding autophagosome-engulfed viroplasms, leading to curtailed RV progeny yield. Other host contributors which aid in the RV morphogenesis within viroplasms are subunits (ATP5B, ATP5A1, ATP5O) of the mitochondrial ATP synthase holoenzyme that re-locate to viroplasms and interact with the 3′ UTR consensus of RV RNAs (5′-UGUGACC-3′). Potential involvement of an intermediate protein has been speculated to facilitate the ATP synthase-RV RNA 3′ UTR association. Chemical inhibitors targeting ATP synthase such as Isoapoptolidin, Venturicidin, and BDM antagonize RV progeny yield.

Similar to RBPs and hnRNPs, many ER chaperones have been reported to be redistributed within or around viroplasmic puncta and interact with specific viroplasmic proteins in RV infected cells (**Figure 5**) (Maruri-Avidal et al., 2008). RV replicates within the host cellular cytoplasm and neither alters the ER membrane morphology nor harnesses the ER resident molecular chaperones for genome replication, questing the implications of such re-programming (Ravindran et al., 2016). More precisely, glucose regulatory protein 94 (GRP94) and 78 (GRP78) have been observed to co-localize with viroplasms (**Figure 4**). For GRP94, RV infection was shown to enhance its protein level and an interaction has been revealed with NSP5, NSP4 as well as VP7). However, transient knockdown of GRP94 did not lower virus infection (Xu et al., 1998; Maruri-Avidal et al., 2008), questioning the relevance of GRP94’s viroplasmic localization. Interaction of GRP78 was found with VP4, VP7 and depletion of this chaperone compromised the production of infectious viral progeny, suggesting its importance in the assembly of mature RV particles (Maruri-Avidal et al., 2008; Zambrano et al., 2011). Other ER chaperones such as Erp57, Calreticulin, and protein disulfide isomerase (PDI) have also been found to get relocated to the proximity of viroplasms in RV infected cells (**Figure 5**). Erp57 depletion did not sensitize RV infection. However, for both PDI and Calreticulin, interaction with VP7 and NSP4 (only for PDI) was observed and both of them positively control the formation of disulfide bonds on VP7 residing on TLPs, justifying compromised yield of infectious progeny in absence of them (Maruri-Avidal et al., 2008; Zambrano et al., 2011). Of relevance, caspase8-dependent co-translocation of Erp57 and Calreticulin from ER to plasma membrane leading to immunogenic cell death (ICD) has been reported for several viruses (Panaretakis et al., 2009; Galluzzi et al., 2010). As caspase8 activation occurs in the late hours of RV infected cells (Martin-Latil et al., 2007; Mukhopadhyay et al., 2022), viroplasmic sequestration of these two chaperones might also provide an active strategy adopted by RV to evade ICD.

Another recent study demonstrated a high-affinity interaction between ATP5B, a core subunit of the mitochondrial ATP synthase, and Group A RV 3′ untranslated region (UTR) consensus (5′-UGUGACC-3′) (Ren et al., 2019). Confocal microscopy affirmed co-localization of ATP5B with the RV 3′ UTR probe within viroplasms of RV infected cells (**Figure 5**). In addition to ATP5B, two other subunits of the ATP synthase complex, ATP5A1, and ATP5O, were also identified as bonafide RV 3′ UTR interactors (**Figure 5**). The functional significance of such cellular ATPase machinery redistribution from mitochondria to RV viroplasms has remained elusive. ATP5B depletion through RNAi or chemical inhibition (by isoapoptolidin, venturicidin, BDM) heavily restricted RV progeny yield by sensitizing late stage of RV life cycle events such as viral genome assembly (**Figure 5**). Therefore, ATPase-driven energy might be critical to foster viral genome packaging. However, the failure of ATP5B to shift the mobility of RV 3′ UTR consensus in an electrophoretic mobility shift assay implies a possible indirect interaction through the involvement of intermediate candidates such as VP1 which accumulates during infection and also has a high affinity for the consensus (Ren et al., 2019).

Host cellular autophagic machinery has been proved to be crucial for DLPs within viroplasms to initiate their morphogenesis (Crawford et al., 2012; Crawford et al., 2019). Macroautophagy is a host cellular catabolic process whereby cargos such as damaged organelles, long-lived proteins, and intracellular pathogens are encaged by double-membrane-bound vesicles called autophagosomes and subsequently channeled through an elaborate intracellular membrane trafficking pathway to lysosomes for degradation of the engulfed contents. In RV infected cells, autophagy is induced; however, the autophagic isolation membranes have been reported to be hijacked from being directed to the canonical lysosomal degradation pathway to ultimately facilitate ER-to-viroplasm transportation of viral proteins NSP4 and VP7 for the production of progeny TLPs (**Figure 5**) (Crawford et al., 2012; Crawford et al., 2019). Agreeably, inhibition of autophagy sensitized RV replication and progeny yield (Crawford et al., 2012; Arnoldi et al., 2014; Mukhopadhyay et al., 2019a). More precisely, the presence of NSP4 and VP7 to reside around autophagosome-engulfed viroplasms was proved to be heavily sensitive to autophagosome inhibition (Crawford et al., 2012). A recent study has revealed the mechanistic detailing of how ER-derived autophagy isolation membranes are redirected in Coat protein complex II (COPII) vesicles from Golgi-apparatus (which are the canonical destination of ER-derived COPII vesicles) to DLPs within viroplasms. In brief, NSP4 exits the ER in COPII vesicles by interacting with the COPII cargo binding protein Sec24. Subsequently, the COPII vesicles are hijacked by the RV-induced LC3 II positive autophagic membranes possibly via the NSP4-LC3 interaction, and the NSP4/LC3 II-containing membranes get redirected to viroplasms (**Figure 5**). COPII vesicle protein Sec31 was reported to interact with both NSP2 and NSP5; however, its exact function in viroplasms has remained unaddressed (Dhillon et al., 2018). Interfering with the COPII vesicle formation/release from ER (either by inhibiting Sar1, a small GTPase regulating the initiation of COPII vesicle formation, through overexpression of its dominant-negative GDP-restricted form or by RNAi-mediated silencing of CK-II which phosphorylates Sec31, an outer coat protein around COPII vesicle) abrogated NSP4 translocation around viroplasms, leading to reduced production of infectious TLPs (Crawford et al., 2019).

## Concluding remarks

RV viroplasms are phase-separated inclusion bodies within the host cellular cytosol where local concentrations for many viral components are high enough to facilitate RV biological processes such as viral genome replication, transcription, and early morphogenesis. They also serve as the safe house for the viral dsRNA population which would otherwise have triggered antiviral host responses. During RV infection, replicative or transcriptive viral dsRNA intermediates may succumb to processing by the host cellular RNAi machinery, yielding virus-derived small interfering RNAs (viRNAs) which might potentially direct viral RNA cleavage and attenuated viral replication. As a countermeasure, RV was found to trigger proteasomal degradation of AGO2, the catalytic effector of the siRNA‐mediated RNAi in mammalian cells, during the early hours of infection, leading to the loss-of functionality of siRNA-based RNAi (Mukhopadhyay et al., 2019b). Going by the notion of viroplasms serving as a safe-house for RV RNAs is also the observation that RNAi-based silencing of NSP1 and NSP3 expression did not sensitize RV genome replication and virion assembly as a whole (Silvestri et al., 2004; Montero et al., 2006). This can be explained by the presence of two distinct and non-exchangeable pools of (+)RNAs—one siRNA-sensitive pool directing translation and the other siRNA-resistant pool guiding dsRNA synthesis— within infected cells. Therefore, the RV (+)RNAs which are used as templates for dsRNA synthesis have most likely originated from the transcriptionally active DLPs within viroplasms. This also partially justifies the poor incorporation of exogenously transfected viral (+)RNAs into the viral safe-houses of infected cells (Silvestri et al., 2004).

Similar to the viral components, viroplasm-associated host proteome is also selective. In most cases, manipulating with the viroplasm associated host components resulted in alteration in the RV progeny yield, vouching for the significance of these host factors in RV infection. However, given that RV life cycle is a multi-step, sequential process where every step depends on the successful completion of the preceding step, the multi-faceted host-viroplasm interaction dynamics have to be interpreted with utmost caution. For example, interfering with the viral entry or other events associated with the initial unmasking of the TLPs may reduce the number of viroplasms formed and promote delayed viroplasm kinetics simply because of the reduced initial viral load. This has to be differentiated from the instances where viral entry is not impeded but viroplasm nucleation is inhibited because of the interference with certain other host determinants such as CK1α, proteasome or LDs (Cheung et al., 2010; Contin et al., 2011; Criglar et al., 2018). Similarly, perturbation of the cytoskeletal network and MRN-ATM-Chk2 branch of the DDR did not sensitize viroplasm nucleation but compromised subsequent viroplasm maturation steps (Eichwald et al., 2012; Sarkar et al., 2020). Interestingly, all these perturbations culminated in the reduction of viral progeny yield despite targeting viroplasm dynamics at different stages. On another cautionary note, the sensitivity of viroplasms in response to a host-targeted small molecule might also be because of a direct effect of the small molecule on viral proteins and viroplasmic architecture and independent of the host target itself. Viroplasmic destabilization observed in presence of RNA polymerase III inhibitor ML-60218 occurs independent of the polymerase inhibitory activity of the small molecule but as a result of the inhibitor’s direct effect on the oligomeric assembly of VP6 trimers (Eichwald et al., 2018). Therefore, the importance of the host factors in regulating viroplasm dynamics needs to be ascertained with more targeted, complementary approaches such as RNAi-based loss-of-functions (Contin et al., 2011; López et al., 2011; Sarkar et al., 2020), clonal overexpression-based gain-of-functions, or loss-of-target sensitivity assay (where removing the host target by secondary means abolishes the activity of the small molecule on RV) (Patra et al., 2019). For most viroplasm-relocated host proteins, the exact molecular mechanism behind the relocation and the exact significance of such redistribution are not dissected, opening many avenues for future research. With the advent of rRVs and proximity-based labelling approaches such as biotinylation, it would be interesting to address the differential composition of host proteome that associate with RV viroplasms at different stages of the RV life cycle.

## Author contributions

PC, SB, PS, UP wrote the manuscript. PC, SB, PS prepared the figures. MC-S and UP edited the manuscript. MC-S provided critical suggestions. All the authors approve the final version of the manuscript.

## Funding

MCS and PC are supported by ICMR. SB and PS are receiving fellowship from UGC and DST respectively.

## Acknowledgments

We acknowledge the contributions of all the research personnel whose rigorous scientific works on host-rotavirus interaction enriched this review. We also acknowledge the research contribution of those scientists whose works were relevant yet exempted from this review because of space constraints. All current members of the Division of virology, ICMR-NICED, Kolkata are deeply acknowledged for their critical evaluation of the manuscript. The contribution of BioRender (through a paid subscription) is also acknowledged for preparing the figures used in the manuscript.

## Conflict of interest

The authors declare that the research was conducted in the absence of any commercial or financial relationships that could be construed as a potential conflict of interest.

## Publisher’s note

All claims expressed in this article are solely those of the authors and do not necessarily represent those of their affiliated organizations, or those of the publisher, the editors and the reviewers. Any product that may be evaluated in this article, or claim that may be made by its manufacturer, is not guaranteed or endorsed by the publisher.
